# Efficacy of Multimodal Sensory Therapy in Adult Acquired Brain Injury: A Systematic Review

**DOI:** 10.1007/s11065-022-09560-5

**Published:** 2022-09-02

**Authors:** Michael Francis Norwood, Ali Lakhani, David Phillip Watling, Chelsea Hannah Marsh, Heidi Zeeman

**Affiliations:** 1 The Hopkins Centre, Menzies Health Institute Queensland, Griffith University, University Drive, Meadowbrook, QLD 4131 Australia; 2 The School of Psychology and Public Health, La Trobe University, 360 Collins St, Melbourne, VIC 3000 Australia; 3 Australian Institute for Suicide Research and Prevention, School of Applied Psychology, W.H.O Collaborating Centre for Research and Training in Suicide Prevention, Griffith University, Brisbane, 4122 Australia; 4 School of Applied Psychology, Griffith University, Gold Coast, QLD 4222 Australia

**Keywords:** Sensory environment, Stroke, Brain injury, Sensory stimulation, Systematic review, Coma

## Abstract

Adults who experience an acquired brain injury often experience disorders of consciousness, physical difficulties, and maladaptive behaviours. Multimodal sensory therapy may benefit brain injured patients, however the extent this therapy can facilitate rehabilitation is not well understood. This systematic review aimed to synthesize multimodal sensory therapy research for adults affected by acquired brain injury. PRISMA guidelines were followed and searches for work published up until July 2021 were undertaken in 5 databases, finding 1054 articles. 43 articles were included in the study. Results describe 29 studies related to coma following an acquired brain injury and 14 to no coma studies (mostly stroke). Multimodal sensory therapy was mostly used as a coma arousal technique following traumatic brain injury, finding positive effects. Multimodal sensory therapy was less applied in stroke, no coma rehabilitation, where most studies found improvement in somatosensory sensation and motor control in an affected limb. In several no coma studies, effects were maintained after several months. The most common senses stimulated in coma studies were audio (*N* = 30), tactile (*N* = 28), visual (*N* = 26), olfactory (*N* = 22), and gustatory (*N* = 17), while the most common senses stimulated in stroke, no coma studies were proprioception (*N* = 7), tactile (*N* = 8), and stereognosis (*N* = 4). Multimodal sensory therapy can be beneficial for patients, especially those in a minimally conscious state or attempting physical rehabilitation following stroke. Negative findings are infrequent in the current literature base. Multimodal sensory therapy appears to be a low-risk intervention with positive outcomes.

## Introduction

Following an acquired brain injury (ABI), as a result of motor vehicle accident, fall, assault or a cerebrovascular event, adults can initially experience disorders of consciousness (i.e., coma), physical difficulties in movement and kinaesthesia, and subsequent maladaptive behaviours such as agitation, aggression or apathy (Deiva et al., 2017). These outcomes are difficult to manage and costly to treat, and may involve a combined approach of psychological, physical or chemical restraint in the most extreme cases (Frasca et al., 2013). In other neuropsychiatric populations a growing body of research indicates that environmental enrichment interventions can promote neuroplasticity and positively impact on disorders of consciousness and subsequent behavioural, cognitive, and social functioning of individuals, such as increased attentional focus (Nithianantharajah & Hannan, 2006; Simpson & Kelly, 2011), increased activity in rehabilitation units (Janssen et al., 2014), and reduced agitation (Fava & Strauss, 2010; Frasca et al., 2013; Kaplan et al., 2006, 2007; McKee et al., 2007). Despite the potential impact of this approach on the rehabilitation of people with ABI, the nature and extent of environmental enrichment therapy and how it could best facilitate positive behavioural adaptation and general rehabilitation is not yet well understood (Li et al., 2020; Pinto et al., 2020).

### Environmental Enrichment and Clinical Application

There is increasing focus on how manipulation of the external environment can influence rehabilitation and recovery following serious injury or illness. Environmental enrichment is the practice of providing external enhancements to a setting that are both complex and novel, thereby increasing environmental engagement and stimulation on behalf of the user (McDonald et al., 2018; Nithianantharajah & Hannan, 2006). The experimental approach, often using rodents, is to encourage exploration, and physical and social activity by enhancing the size of the living space and increasing the quantity of novel objects of various shapes (Benaroya-Milshtein et al., 2004; Zebunke et al., 2013). Benefits of environmental enrichment on sensorimotor and cognitive outcomes are wide ranging (Nithianantharajah & Hannan, 2006; Simpson & Kelly, 2011) and animal studies have reported cerebral changes at a morphological and molecular level (Alwis & Rajan, 2014; Mesa-Gresa et al., 2013; Rosenzweig et al., 1978; Sozda et al., 2010).

At a cellular level, environmental enrichment affects neuronal functioning through a range of interactions, leading to positive changes in sensorimotor and cognitive behaviour, making environmental enrichment an ideal treatment approach for ABI such as traumatic brain injury (TBI) (Alwis & Rajan, 2014). TBI fundamentally alters neuronal functioning in the sensory cortices (Ding et al., 2011; Hall & Lifshitz, 2010) and approximately 60% of patients display sensory deficits (Carey, 1995), which Alwis and Rajan (2014) argue contributes to persistent cognitive deficits typically found in patients. Thus, one obvious area for environmental intervention in TBI relates to enrichment of the sensory cortex – this can be achieved through targeted sensory stimulation therapy, aided by environment design input (Gardner et al., 2000). Outcomes from environmental enrichment in rats have been improved by the addition of sensory stimulation (Maegele et al., 2005). For example, in animal studies, the most common sensory stimulation in environmental enrichment is through auditory stimuli (Alwis & Rajan, 2014) and findings include enhanced synaptic transmission in the auditory cortex (Percaccio et al., 2005, 2007).

### Sensory Stimulation

Humans engage in at least five sensory experiences, namely touch, taste, smell, sight, and hearing, though there are other sensory modalities that do not receive as much attention (Gardner et al., 2000; Stillman, 2002). Sensory stimulation may occur through environmental design such as is done in environmental enrichment. However, it may also involve direct stimulation of any sensory modality (Karma & Rawat, 2006). Sensory stimulation can be unimodal or multimodal, however, contemporary neuroscience research suggests that sensory modalities more effectively operate in concert with each other (i.e., multimodal) as part of a ‘whole of brain’ response, as opposed to in a unimodal process (e.g., Baier et al., 2006). Indeed, it would seem multimodal approaches to sensory stimulation are more effective than unimodal (Pinto et al., 2020; Zuo et al., 2021).

### Multimodal Sensory Stimulation

Studying sensorimotor recovery following an ABI as a unimodal construct is not justified as the idea of ‘modality-specific’ cortices is no longer prevalent. As Shimojo and Shams (2001) stated, “interaction between modalities is the rule as opposed to the exception in brain function” (pg. 508); the brains cross-modal cortical processing plays a substantial role in day-to-day adaptive behaviour (Shimojo & Shams, 2001). A variety of evidence supports the notion of multi-modularity (Shimojo & Shams, 2001). For example, following an ABI, improved sensorimotor functioning appears to reflect behavioural compensation from unimpaired alternate modalities rather than functional recovery from impaired brain regions (Jadavji et al., 2006; Rose et al., 1993). Furthermore, visual dependence displayed by stroke patients does not mean other sensory modalities are neglected as stroke patients also rely on visual, proprioceptive and vestibular information for posture control (Bonan et al., 2016). Indeed, environmental enrichment is limited when tasks involved are unimodal (Rose, 1988) and Zuo et al. (2021) found that family centred sensory stimulation was more effective when multimodal sensory approaches are taken. Generally, multimodal sensory approaches are economical, simple, stimulate a number of senses (Park, 2016), and are commonly delivered by nurses or therapists (Zuo et al., 2021). For example, Megha et al. (2013) describe a multimodal sensory approach that included speaking to the patient and reading (auditory), displaying photographs (visual), presenting favourite aromas (olfactory), and applying different materials to the patient’s arm (touch).

Yet, despite the reasoning for the multimodal sensory stimulation approach, the research literature relating to multimodal sensory stimulation and its relationship to human ABI rehabilitation and environmental design is limited. Previous systematic and scoping reviews shed some light on the evidence. However, these reviews used limited search terms (Li et al., 2020; Padua et al., 2019), limited databases (Cameron et al., 2020; Li et al., 2020; Padua et al., 2019), included unimodal sense therapies (Li et al., 2020; Padua et al., 2019) or found them prevalent in their search results (Pinto et al., 2020), focused on the delivery by family members (Zuo et al., 2021), did not divide outcomes into senses stimulated (Cameron et al., 2020), or focused on methodological characteristics (Pinto et al., 2020).

This systematic review aims to synthesize research evidence relating to multimodal sensory interventions for adults affected by ABI. This review builds on previous reviews by excluding unimodal studies, expanding the search strategy, and extracting data based on injury, actual senses stimulated, and outcomes reported. The specific research question was (a) What is the influence of multimodal sensory therapy on cognitive, physical or behavioural functioning on adults affected by ABI?

## Method

### Search Strategy and Selection Criteria

In accordance with PRISMA guidelines (Moher et al., 2009), a systematic review was undertaken of the published research literature relating to multimodal sensory interventions for people with ABI up to and inclusive of July 2021. The databases CINAHL, PubMed, ProQuest, PsychInfo, and Web of Science were used. Search strategy included terms around sensory, brain injury, and therapy; these can be found in Appendix 1. Inclusion criteria were adult populations (aged over 18 years) receiving multimodal sensory stimulation in a rehabilitation or treatment context. Peer-reviewed and English language studies with any publication date were included. Exclusion criteria were participants with co-diagnosis of autism or intellectual disability and dementia populations. The review was registered with the Centre for Reviews and Dissemination (UK) in October 2016 and PROSPERO International prospective register of systematic reviews in 2016 (Zeeman et al., 2016).

### Data Extraction and Quality Assessment

Initial title and abstract checks were completed by a single reviewer. Two researchers then reviewed the full-text showing overall agreement of 92%. Cohen’s K suggested substantial agreement between the two researchers, *ĸ* = .643 (95% CI, 430–.856). Discrepancies around final inclusion were resolved on agreement from all authors. The following data was extracted from the selected studies: author, year of publication, country, study design, sample, treatment conditions, sensory modalities tested, intervention description, outcome measures, and main results. Quality of studies and risk of bias were assessed using the McMaster quantitative rating scale (Law et al., 1998). The rating form is comprised of 8 overarching criteria containing descriptive and yes–no questions for respondents to answer. 15 yes–no response questions were rated in the present review where a “yes” response was designated with a 1 and a “no” or “not addressed-unclear” response with a 0. McMaster’s quantitative rating scale divides bias into 3 main areas, (a) sample biases, (b) measurement biases, and (c) intervention biases and this is recorded qualitatively. Two researchers independently rated studies. For moderation, a sub-sample of studies were cross-checked and any areas of uncertainty where rectified.

To evaluate the quality of included studies further, studies will also be categorised into international guidelines on evidence level (I, II, III, IV, V).

## Results

### Screening and Study Selection

**Fig. 1:**
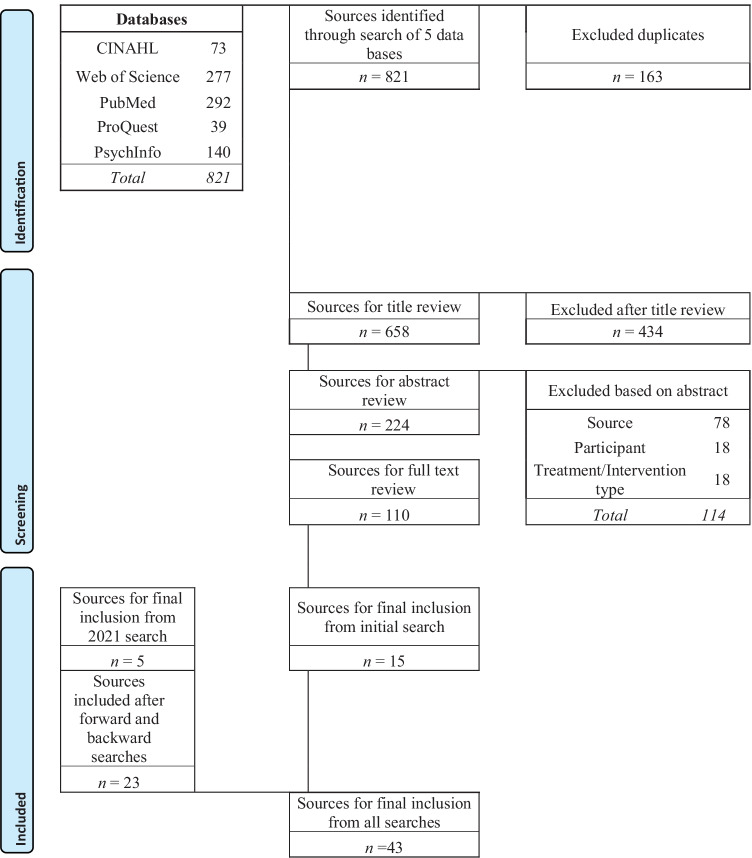
Screening and study selection process

A total of 821 articles were found in June 2017 with 15 included and 23 included through forward and backward searching. A second search was carried out in July 2021 finding 233 additional papers with 5 finally included. The number of articles at final inclusion totalled 43. Papers were screened in the following steps, (a) duplicate search, (b) title review, (c) abstract review, (d) full text review and, (e) backward and forward searches. See Fig. 1 for detailed flow chart.

### Level of Evidence

Study selection included 11 randomized-control trials, 5 quasi-experimental prospectively controlled study, 5 pre-post test or retrospective control group studies, 1 case controlled study, 3 case studies, and 18 observational studies without a control group.

The number and types of studies at each level of evidence can be seen in Table 1 below.

**Table 1:** Number and types of studies at each evidence level

Levels of Evidence - Effectiveness		Number of Sources
Level 1 – Experimental Designs	Level 1.a – Systematic review of Randomized Controlled Trials (RCTs)	
	Level 1.b – Systematic review of RCTs and other study designs	
	Level 1.c – RCT	11
	Level 1.d – Pseudo-RCTs	
Level 2 – Quasi-experimental Designs	Level 2.a – Systematic review of quasi-experimental studies	
	Level 2.b – Systematic review of quasi-experimental and other lower study designs	
	Level 2.c – Quasi-experimental prospectively controlled study	5
	Level 2.d – Pre-test – post-test or historic/retrospective control group study	5
Level 3 – Observational – Analytic Designs	Level 3.a – Systematic review of comparable cohort studies	
	Level 3.b – Systematic review of comparable cohort and other lower study designs	
	Level 3.c – Cohort study with control group	
	Level 3.d – Case – controlled study	1
	Level 3.e – Observational study without a control group	19^*^
Level 4 – Observational – Descriptive Studies	Level 4.a – Systematic review of descriptive studies	
	Level 4.b – Cross-sectional study	
	Level 4.c – Case series	
	Level 4.d – Case study	3
Level 5 – Expert Opinion and Bench Research	Level 5.a – Systematic review of expert opinion	
	Level 5.b – Expert consensus	
	Level 5.c – Bench research/ single expert opinion	
Total		44 (43 total sources)

^*^One source reported a study and a pilot study in the same publication, both have been rated on the JBI

### Methodological Quality

The methodological quality assessment of each article is provided in Table 2. The range of quality appraisal scores was 7–15 (out of 15) and the average was 12.27.

**Table 2:** Quantitative studies review form

Study	Purpose clearly stated	Relevant literature reviewed	Sample described in detail	Sample size justified	Informed consent obtained	Reliable outcome measure	Valid outcome measure	Intervention described in detail	Contamination avoided	Cointervention avoided	Statistical significance reported	Appropriate analysis method	Clinical importance reported?	Drop-outs reported	Conclusion appropriate	Total /15
1	1	1	1	1	1	1	1	1	1	1	1	1	1	1	1	15
2	1	0	1	0	1	1	1	1	0	0	1	1	1	1	1	11
3	1	1	1	1	1	1	1	1	1	1	1	1	1	1	1	15
4	1	0	1	1	1	1	1	1	1	1	1	1	1	1	1	14
5	1	1	1	0	0	1	1	0	0	0	0	1	1	1	1	9
6	1	1	1	1	1	1	1	1	1	1	1	1	1	1	1	15
7	1	1	1	0	1	1	1	1	0	1	1	1	1	0	1	13
8	1	0	1	0	1	1	1	0	1	1	1	1	0	1	1	10
9	1	1	1	1	1	1	1	1	1	1	1	1	1	1	1	15
10	0	1	1	0	1	0	0	0	1	1	0	0	1	0	1	7
11	1	1	1	1	1	1	0	1	1	1	1	1	1	0	1	13
12	1	0	1	1	1	1	1	0	1	0	0	0	0	1	0	8
13	1	1	1	0	1	1	1	1	1	1	1	1	1	1	1	14
14	1	1	1	1	1	1	1	1	1	1	0	0	0	1	1	12
15	1	1	1	0	1	1	1	1	1	1	1	1	1	0	1	13
16	1	1	1	1	1	1	1	1	1	1	0	1	1	1	1	14
17	1	1	1	0	1	1	1	1	1	1	1	1	1	1	1	14
18	0	1	1	0	1	1	1	1	1	1	1	1	1	0	1	12
19	1	1	1	1	1	1	1	1	1	1	0	0	1	0	1	12
20	1	1	1	0	1	1	1	1	1	1	1	1	1	0	1	13
21	1	1	1	1	1	1	1	1	1	1	1	1	1	0	1	14
22	1	1	0	0	1	0	0	0	1	1	0	0	1	0	1	7
23	1	0	0	0	1	1	1	1	1	0	1	1	1	0	1	10
24	1	1	1	1	1	1	1	1	1	0	1	1	1	1	1	14
25	1	1	1	0	1	1	1	1	1	1	1	1	1	0	1	13
26	1	1	1	1	1	1	1	1	1	1	1	1	1	0	1	14
27	1	1	1	1	1	1	1	1	1	1	1	1	1	1	1	15
28	1	1	1	0	1	0	0	1	1	1	1	1	0	0	1	10
29	1	1	1	0	1	1	1	1	1	1	1	1	1	1	1	14
30	1	1	0	0	1	1	1	1	1	1	1	1	1	1	1	13
31	1	1	1	1	1	1	1	1	1	1	1	1	1	1	1	15
32	1	1	0	0	1	1	1	1	1	1	1	0	0	1	1	11
33	1	1	1	1	1	1	1	1	1	0	1	1	0	1	1	10
34	1	1	1	1	1	1	1	1	1	0	1	1	1	1	1	14
35	1	1	1	0	1	0	1	1	1	1	1	1	1	0	1	12
36	1	1	1	0	1	1	1	0	1	1	0	1	1	0	1	11
37	1	1	1	1	1	1	1	1	1	1	1	1	1	1	1	15
38	1	1	1	0	1	1	1	0	1	1	1	1	1	1	1	13
39	1	1	1	0	1	0	0	1	0	1	1	1	1	0	1	10
40	1	1	1	0	1	0	0	1	0	1	1	1	1	0	1	10
41	1	1	1	0	1	1	0	0	0	1	1	1	1	0	1	10
42	1	1	1	0	1	0	0	1	0	1	1	1	1	0	1	10
43	1	1	1	1	1	0	0	1	1	1	1	1	1	1	1	13

For each criterion: 1 = criterion met; 0 = criterion not met or unclear if met

1. Abbasi et al. (2009); 2. Attwell et al. (2019); 3. Bonan et al. (2016); 4. Byl et al. (2008); 5. Canedo et al. (2002); 6. Carey et al. (1993); 7. Cheng et al. (2018); 8. Deiva et al. (2017); 9. de Diego et al. (2013); 10. de Jersey (1979); 11. Di Stefano et al. (2012); 12. Doman et al. (1993); 13. Gomez et al. (2016); 14. Hall et al. (1992); 15. Heine et al. (2017); 16. Helliwell (2009); 17. Dogru Huzmeli et al. (2017); 18. Johnson et al. (1993); 19. Kaewsriwong et al. (2015); 20. Kater (1989); 21. Keller et al. (2007); 22. Grüner and Terhaag (2000); 23. Lynch et al. (2007); 24. Mandeep (2012); 25. Megha et al. (2013); 26. Mitchell et al. (1990); 27. Moattari et al. (2016); 28. Noda et al. (2004); 29. Oh and Seo (2003); 30. Pierce et al. (1990); 31. Poza et al. (2013); 32. Rader et al. (1989); 33. Sargolzaei et al. (2017); 34. Sedghi and Ghaljeh (2020); 35. Smania et al. (2003); 36. Talbot and Whitaker (1994); 37. Urbenjaphol et al. (2009); 38. Wijnen et al. (2006); 39. Wilson et al. (1991); 40. Wilson et al. (1993); 41. Wilson et al. (1996); 42. Wood et al. (1993); 43. Yekutiel and Guttman (1993)

### Risk of Bias

Identified and potential risks of bias in the main areas of sampling, measurement and treatment for each study can be found in Appendix 2. Biases were scored on which direction they would skew the results (i.e., would they favour treatment/experimental hypothesis or control/null hypothesis). Some common risks of bias were unavoidable and existed across almost all studies. This included sampling biases, where recruitment was largely on a volunteer basis and with family involvement; measurement biases, which centred around raters being non-blinded or the rater was unknown (for studies in a hospital blinding measurement would require more resources); and treatment biases, regarding co-intervention, due to the severe medical needs of patients, and a lack of consistency in who the treating therapist was, and what the treatment time was throughout the day. An attention bias, where people are aware of the study so perform better or give favourable responses, was likely present in many studies. However, this was also largely unavoidable given the presence of families and their involvement in many studies as delivering the intervention or rating the outcome. Finally, case studies were not usually clear on how they recruited or if they excluded patients, which may have created a reporting bias. Altogether, this resulted in a small amount of bias favouring the intervention or experimental hypothesis in a large majority of coma studies (19/28; 76%) and just over half no coma studies (7/15; 54%). Bias was found to be negligible in 6/28 (21%) coma studies and 7/15 (54%) no coma studies with no studies biasing the control group or null hypothesis.

Almost all studies had at least one bias where there was not enough information to judge the direction of bias (e.g., it was often unknown who collected data, if they were blinded, and if the same person delivered the treatment across the study.) but in only 4/43 studies was it not possible to give an overall judgement of direction.

### Study Characteristics

11 studies were from Europe, 7 were from Asia, 6 were from the UK, 6 were from the U.S.A, 4 were from Australia, 2 were from Canada, 5 were from the Middle East, 1 was from Turkey and 1 was cross-cultural (China and Italy).

The studies were heterogenous in terms of the assessment criteria and outcome measures, although there was commonality in coma studies that primarily measured improvement on the Glasgow Coma Scale (GCS), and to a lesser extent the Rancho Los Amigos Level of Cognitive Function Scale (RLA) and the Western Neuro Sensory Stimulation Profile (WNSSP). Other measures included the Functional Impairment Measure (FIM), Mini-Mental State Exam (MMSE), Semmes–Weinstein, Wessex Head Injury Matrix (WHIM), Glasgow Outcome Scale (GOS), posture, Fugl Meyer Assessment (FMA), Rivermead Assessment of Somatosensory Performance (RASP), Coma Recovery Scale (CRS) and Coma Recovery Scaled-Revised (CRS-R), Richmond Agitation and Sedation Scale (RASS), Sensory Modality Assessment and Rehabilitation Technique (SMART), texture discrimination, sensory assessments and various neurological or behavioural measures such as eye opening or tracking, electroencephalogram (EEG), fMRI, or heart rate. Table 3 presents the main characteristics and results of articles.

**Table 3:** Main Characteristics and Results of Included Studies

**First Author (year), Country**	**Study Design**	**Outcome Measurement**	**Positive (+) and/or Negative (-) Findings**	**Statistically Significant Results Reported**	**Sensory Modalities Tested**	**Intervention/Test duration**
*Coma Studies (n* = *29)*						
Abbasi et al. (2009), Iran	RCT	GCS	+	Yes	A,T,Affect	Daily, 15 min for 6 days
Attwell et al. (2019), Switzerland	Prospective Cross over	CRS-R	+	Yes	A, V, T, Ph	2 × 25 min session Indoor & Outdoor - randomized
Canedo et al. (2002), U.S.A	Case study	GCS, RLA	+	No	A,V,G,T	< 3 months
Cheng et al. (2018), China/Italy	Single-case; ABAB Withdrawal	CRS-R	-/+	Yes	A, T, O, V, G	6 × 20 min over 4 weeks, A Phase = Rehabilitation
Deiva et al. (2017), India	Pre-Post	CRS-R, GCS	±	Yes	A, V, O, T	2 weeks
Di Stefano et al. (2012), Italy	Pre-Post	WHIM	+	Yes	A,V,O,T	Daily, 5 weeks
Doman et al. (1993), U.S.A	Pre-Post	GOS	+	No	A,V,O,G,T	Hourly, 6 days/week > 4 months
Hall et al. (1992), Canada	Pre-Post	WNSSP, GCS, RLA, Rader Scale	+	No	A,V,O,G,T	Once daily, 30 min, weekdays for two weeks. 2 or 3 rotations per participant
Johnson et al. (1993), UK	RCT	GCS, GSR, HR	-/+	Yes	A,V,O,G,T	1 h daily
Kaewsriwong et al. (2015), Thailand	Case study	GCS, RLA, SMART	+	No	A,V,O,G, T,K	Daily, < 6 months
Kater (1989), U.S.A	Pre-Post	GCS, EII, Cog Assess	+	Yes	A,V,O,G,K,C	2 × day 45 min for 6 days over 1–3 months
Keller et al. (2007), Germany	Pre-Post	EEG	+	No	A,T	Insufficient detail
Grüner and Terhaag (2000), Germany	Pre-Post	GOS, Neurological condition	+	No	A,V,O,G,T,P	2, 1 h sessions daily for around 9 days. 10 min rest 10 min stimulation pattern
Megha et al. (2013), India	RCT	GCS, WNSSP, eye tracking, arousal	+	Yes	A,V,O, G, T,	Group A: 5 × day 20 min for 5 weekdays over 2 weeks; Group B: 2 × day 50 min for 5 weekdays over 2 weeks
Mandeep (2012), India	RCT	GCS, CRS	+	Yes	A,V,T,K	Each sense stimulated twice a day (approx. 30 s each) for 2 weeks
Mitchell et al. (1990), UK	Pre-Post	GCS	+	No	A,V,O, G, T,	2 × day over 4 weeks
Moattari et al. (2016), Iran	RCT	GCS, RLA, WNSSP	+	Yes	A,V,O,T	2 × day over 7 days
Noda et al. (2004), Japan	Pre-Post	PVS	+	Yes	A,T,K	5 min × 3 over 30 min
Oh and Seo (2003), Republic of Korea	Observational	GCS	+	No	A,V,O,G,T,Ph	2 × day over 7 days, 4 weeks over 4 months
Pierce et al. (1990), Australia	Pre-Post	GCS	-	No	A,V,T	8 h daily, until patient accepted for conventional therapy
Rader et al. (1989), U.S.A	Observational	Eye opening	+	No	A,V,O,G, T,	30-45 min
Sargolzaei et al. (2017), Iran	RCT	SMART	-/+	Yes	A,V, O, G, T, Ph	14 weeks x 90 min daily
Talbot and Whitaker, (1994), Canada	Pre-Post	GCS, DRS, LCFS, CNCS, Freeman Questionnaire	+	No	A,V,O,T,P	Insufficient detail; 4 intervention phases, participants received at least 2; Some indication intervention lasted for 24 months
Urbenjaphol et al. (2009), Thailand	RCT	GCS, SMART	±	Yes	A,V,O,G,T	30 min every 2 h daily over 2 weeks
Wijnen et al. (2006), The Netherlands	Pre-Post	WNSSP, DRS, HR GOS-E,	+	Yes	A,V,O,T	15 min
Wilson et al. (1991), UK	Pre-Post	Eye opening, Body movement	±	Yes	A,V,O,G,T	10 min daily
Wilson et al. (1993), UK	Observational	Eye opening, Body movement	±	Yes	A,V,O,G,T	Daily over 15 days, < > 3 months
Wilson et al. (1996), UK	Cohort	Eye opening, Body movement, Activity Engagement, Vocalisation	+	Yes	A,V,O,G,T	3-week blocks, 2 × daily; 1–8 sets of treatment blocks needed
Wood et al. (1993), U.S.A	Pre-Post	GCS, SRH, RLA	+	Yes	A,V,O,T	5 s stimulus, 10 s break; 5 trials per modality. Unclear how long each session took
*No Coma Studies (n* = *14)*						
Gomez et al. (2016), Spain	Pre-Post	EEG	±	Yes	A,V	18 min
de Diego et al. (2013), Spain	RCT	FMA, SIS-16, MAL, ST	+	Yes	P, T	16 one hr sessions over 8 weeks
Poza et al. (2013), Spain	Pre-Post	EEG	+	Yes	V,A	18 min
Smania et al. (2003), Italy	Observational	Task performance	+	Yes	V,T,W,P,S	Insufficient detail – 30 training sessions completed
Carey et al. (1993), Australia	Pre-Post	Texture discrimination	+	No	T, P	10 sessions, 15–30 min
Yekutiel and Guttman (1993), Israel	Pre-Post	Sensory training	+	Yes	T, P, S	45 min over 6 weeks
de Jersey (1979), Australia	Pre-Post	Sensory Assessments	-/+	No	T, Temp, Pressure	1–2 min
Heine et al. (2017), France	Pre-Post	CRS-R	±	Yes	A,O,N	4 sessions 20 min over 4 weeks
Bonan et al. (2016), France	Pre-Post	Posture: Optokinetic and galvanic vestibular stimulation	+	Yes	V,P,K	15 s rest, 35 s right side stimulation, 3 min rest, 35 s left side stimulation
Byl et al. (2008), U.S.A	RCT	FIM, FMC, Strength	±	Yes	K, S, G	12–72 h up to 6 weeks
Lynch et al. (2007), Australia	RCT	Semmes–Weinstein	-/+	Yes	T, Temp	10 × 30 min over 2 weeks
Helliwell (2009), UK	Case study	RASP, FIM	-	No	T, P, S	Weekly over 3 weeks
Dogru Huzmeli et al. (2017), Turkey	RCT	MMSE, TIS	-	Yes	T, P	10 × 45 min over 2 weeks
Sedghi and Ghaljeh (2020), Iran	Pre-Post	RASS	-/+	Yes	A, T	7 × 10 min over 1 week, stimulation measured pre stimulation and 30 min post

### Sample Characteristics

The sample sizes ranged from 1 to 233 participants. 28 studies related to coma following ABI (mostly TBI) and 1 to disorder of consciousness following stroke; these have been grouped together and labelled ‘coma studies.’ An additional 3 studies related to TBI no coma and 10 to stroke no coma; these have been grouped together and labelled ‘no coma’ studies. There were no studies identified relating to MSST for adults with multiple sclerosis, cerebral palsy, or spinal cord injury. There were no studies identified relating to use of MSST in people who were in post-traumatic amnesia (PTA) or post-PTA and medically stable following acquired or traumatic brain injury. For more details on sample characteristics see Table 4.

**Table 4:** Participant Characteristics in Included Studies

**Coma studies**	**N**	**Patient Population**	**Sex (% male)**	**Mean Age**	**Time since injury**	**Ethnicity**	**Affected side (% right)**
Abbasi et al. (2009)	50 (25 in intervention)	TBI	88%	30.4	Newly admitted	Iranian	N/A
Attwell et al. (2019)	15	TBI	73%	52.6	25.2 days	Swiss	N/A
Canedo et al. (2002)	2	TBI (mixed)	50%	24 & 45	3 months	Caucasian & African American	Bilateral
Cheng et al. (2018)	29	TBI (mixed)	65%	48	less than a year to 10 years	Chinese or Italian	N/A
Deiva et al. (2017)	30 (15 intervention)	TBI	77%	N/A	72 h	Southern Indian	N/A
Di Stefano et al. (2012)	11	TBI/Stroke/Anoxic BI	N/A	30.75	5.75 months	Italian - Unknown	N/A
Doman et al. (1993)	233	TBI	67%	N/A	6 months average (1–12 months)	N/A	N/A
Hall et al. (1992)	6	TBI	83%	37.5	15.8 days	N/A	17%
Johnson et al. (1993)	14 (7 intervention)	TBI	100%	27.7	< 48 h	N/A	N/A
Kaewsriwong et al. (2015)	2	TBI	50%	27 and 19	N/A	N/A	0
Kater (1989)	30 (15 intervention)	TBI	60%	28	< 2 weeks	N/A	N/A
Keller et al. (2007)	18	TBI/Hypoxia	61%	39.3	18 months	Germany - unknown	N/A
Grüner and Terhaag (2000)	89 or 16	BI	N/A	43.6	< 48 h	Germany - unknown	N/A
Megha et al. (2013)	30	TBI	N/A	39.3	7.7 days	Indian	N/A
Mandeep (2012)	30 (15 in intervention)	TBI	N/A	N/A	N/A	Indian	N/A
Mitchell et al. (1990)	24 (12 intervention)	TBI	83%	22.3	7.9 days	UK	25%
Moattari et al. (2016)	60 (20 in each of two intervention groups)	TBI	82%	37	4 days	Iranian	N/A
Noda et al. (2004)	26	TBI/Stroke	66%	38.5	N/A	Japan	
Oh et al. (2003)	5	TBI	100%	50.2	< 3 months	South Korean	N/A
Pierce et al. (1990)	31	TBI	67%	24	N/A	N/A	N/A
Rader et al. (1989)	6	TBI	66%	3 patients 18–19 yrs + 3 40 -55	15.5 months	N/A	0
Sargolzaei et al. (2017)	80	Stroke	45%	66.2	N/A	Iranian	37.50%
Talbot and Whitaker (1994)	8	BI	71%	N/A	N/A	N/A	N/A
Urbenjaphol et al. (2009)	40 (20 intervention)	TBI	70%	33.4	6.8 days	Thai	15%
Wijnen et al. (2006)	16	TBI	61.10%	21.5	2.3 months	Dutch - Unknown	N/A
Wilson et al. (1991)	3	TBI	100%	15, 36, &41		UK - Unknown	
Wilson et al. (1993)	7	TBI	7 single case studies - all appear to be male			UK - Unknown	
Wilson et al. (1996)	24	TBI/Hypoxia/Other	N/A	28.75	16.2 months	UK - Unknown	N/A
Wood et al. (1993)	15	TBI/SAH	40%	34.6	N/A	N/A	N/A
*No Coma Studies*							
Gomez et al. (2016)	36	TBI/CP	72%	41	> 8 years	N/A	19%
de Diego et al. (2013)	21 (12 in experimental)	Stroke	N/A	61.9	44.7 months	N/A	N/A
Poza et al. (2013)	36 (18 finally included)	TBI	61%	38.4	Majority more than 8 years	Spain - Unknown	22%
Smania et al. (2003)	3	Stroke	50%	51.8	N/A	N/A	75%
Carey et al. (1993)	8	Stroke	87%	49.8	12.8 weeks	N/A	25%
Yekutiel and Guttman (1993)	39	Stroke	65%	64	6.2 years	Israel - Unknown	50%
de Jersey et al. (1979)	20	Stroke	55%	N/A	N/A	N/A	25%
Heine et al. (2017)	13	TBI/Stroke	69%	44.5	30 months	French - Unknown	N/A
Bonan et al. (2016)	35	Stroke	62%	54.1	3 months	N/A	48%
Byl et al. (2008)	45	Stroke	62%	62	2.4	Caucasian	57%
Lynch et al. (2007)	21 (10 in intervention)	Stroke	70.00%	61	48.7 days	N/A	50%
Helliwell (2009)	1	Stroke	0	78	21 days	N/A	0
Dogru Huzmeli et al. (2017)	26 (13 in intervention)	Stroke	69%	53	40.23 months	Turkey - Unknown	92%
Sedghi and Galjeh (2020)	80 (40 intervention)	TBI	60%	40.2	N/A	Iranian	N/A

### Intervention Findings

Information on the main characteristics and outcomes of studies can be found in Table 3. Multimodal sensory exposure was mostly implemented as a coma arousal technique following very severe ABI (commonly TBI). Most coma studies reported only positive changes in level of consciousness (*N* = 21) with six reporting mixed results and one reporting no significant changes. One study reported lower oscillatory waves as measured by EEG and interpreted this as a state of relaxation (Poza et al., 2013). One study reported higher activation in the right middle frontal gyrus, right superior temporal gyrus and bilateral ventro-anterior thalamic nucleus when using fMRI during treatment (Cheng et al., 2018) but urge caution of strong interpretation of these findings given a sample of 3. The most common senses stimulated in coma studies were audio (*N* = 30), tactile (*N* = 28), visual (*N* = 26), olfactory (*N* = 22), and gustatory (*N* = 17). Treatment *doses* varied, and included frequent as well as less frequent exposure over day, weekly and monthly periods. One study concluded that more frequent, less intense exposure is better (Megha et al., 2013).

Secondary use of multimodal sensory stimulation was in stroke, or TBI no coma, rehabilitation. Most studies found improvement in somatosensory sensation and motor control in an affected limb (Carey et al., 1993; de Diego et al., 2013; de Jersey, 1979; Dogru Huzmeli et al., 2017; Smania et al., 2003; Yekutiel & Guttman, 1993). Training was only required for 2 – 8 weeks, although high intensity promoted better outcomes in sensory discrimination and strength (Byl et al., 2008). In several studies, effects were maintained after several months (Carey et al., 1993; Smania et al., 2003). One study reported positive findings in posture and balance (Bonan et al., 2016). Contrastingly, one study found balance was not significantly different than an active control (Dogru Huzmeli et al., 2017). A single person case study found positive effects on proprioception, but without this affecting motor recovery (Helliwell, 2009), and Lynch et al. (2007) found no differences between experimental group and a control group (all of whom improved in posture, gait, or assisted walking). In contrast to coma studies, audio was never the sensory stimulation method used in post-stroke, no-coma rehabilitation studies. The most common senses stimulated were proprioception (*N* = 7), tactile (*N* = 8), and stereognosis (*N* = 4).

## Discussion

This systematic review aimed to synthesise research evidence relating to multimodal sensory interventions for adults affected by ABI, asking the question, what is the influence of multimodal sensory therapy on cognitive, physical or behavioural functioning on adults affected by ABI? This review finds that multimodal sensory stimulation may be a facilitator of arousal in minimally conscious or comatose states following severe TBI; a finding reported in previous reviews (Li et al., 2020). This is a promising finding given that behavioural responses during a minimally conscious state are associated with emergence from this state (Wilson et al., 1996). There is also some evidence that following stroke (no coma), participants presented with enhanced sensations and motor control in affected limbs following multimodal sensory stimulation. Therefore, intervention with patients with different levels of consciousness appears to have different requirements, which led to the exclusion of coma patients on previous reviews (Pinto et al., 2020).

The results of this review suggest practice has been to use more, and a wider variety of senses, for patients in a coma and minimally conscious states, and less but more targeted senses for those recovering from a stroke (no coma). In minimally conscious states it is common for a minimum of four senses to be targeted to improve level of consciousness. Likely due to more targeted treatment needs, stroke patients were commonly stimulated with two or three senses related to effective movement and orientation in a physical environment, such as proprioception, balance, posture and touch. This difference reflects the patients underlying condition. The specific senses stimulated in stroke reflect the focus in stroke rehabilitation of addressing mobility and activities of daily living (Stein et al., 2021). The higher number of senses in minimally conscious states reflects the contemporary neurosciences ‘whole of brain’ approach (e.g., Baier et al., 2006). For example, synchronized communication across several brain regions, of sufficient complexity, is needed to maintain consciousness (Alnes et al., 2021; Deco et al., 2015).

There is little research exploring dosage, a finding consistent with past research (Pinto et al., 2020). Guidance may be found in previous related environmental enrichment research which suggests shorter periods of exposure have limited effect, therefore there is a threshold of exposure needed before benefits are seen (de Witt et al., 2011). The current findings suggest that high frequency stimulation targeting physical movement may promote better outcomes in no coma stroke patients, and that more frequent but less intense stimulation may be beneficial for patients in a coma aiming to improve conscious state. Indeed, following their review of the literature on sensory stimulation for people in a coma after an ABI, Padilla and Domina (2016) also suggest frequent stimulation is more effective. It has also been suggested that stimulation must start early (Padilla & Domina, 2016; Zuo et al., 2021). However, these conclusions are based on limited research and require more investigation before they can be meaningfully suggested.

Related to dosage is the notion of personalisation of sensory stimulation. Preferred music had a greater effect than neutral music on patient’s responsiveness (Heine et al., 2017) and sensory stimulation was improved when delivered by families rather than by clinical staff (Moattari et al., 2016; Sedghi & Ghaljeh, 2020). Cheng et al. (2018) and Sargolzaei et al. (2017) even concluded a priori that multimodal sensory stimulation was better delivered by family members and chose this as part of their intervention group. It is likely that personalised sensory stimulation therapy arouses increased affective responses. For example, music elicits a greater emotional response (Moattari et al., 2016) and music that a patient prefers may result in stronger emotions than neutral music. In essence, personalised approaches may result in more intense, emotion eliciting dosages that may encourage stronger cortical responses. This outcome is supported by research exploring unimodal therapies (e.g., Sullivan et al., 2018; Tavangar et al., 2015; Zuo et al., 2021). For example, a recent review found family-centred sensory stimulation for comatose patients following a TBI was more effective than clinician implemented or routine care (Zuo et al., 2021).

One recent study compared outdoor multimodal sensory stimulation with indoor (Attwell et al., 2019). The outdoor therapy was embedded in a natural setting and the authors found this was more effective than indoor settings. This finding is not surprising, given the differing effect of a green environment on brain activity (Norwood et al., 2019) and the weight of research finding positive effects of green environments on cognitive functions (e.g., Bratman et al., 2012; Kuo et al., 2019). Research suggests the positive effects of natural settings are also stronger in multimodal sensory green environments rather than unisensory. For example, a recent study found natural olfactory stimuli may be more important than natural visual stimuli for stress reduction, leading the authors to conclude that urban planners should consider multimodal sensory stimuli in greenspaces, where current practice prioritises visual stimuli (Hedblom et al., 2019). Another study found that during green exercise, the occlusion of individual sensory stimuli resulted in lower mood than a full sensory experience (Wooller et al., 2015). Indeed, a recent review of studies exploring the effects of simulated nature on human health and cognitive functioning concluded multimodal sensory stimulations were a prime opportunity for research (Browning et al., 2021). If it is practical to complete multimodal sensory stimulation in green environments, then this is recommended. Future research can now explore the specific effects of dosage, specific natural stimuli, and frequency of exposure. In line with the aforementioned, this will likely be more effective if completed by family members and if stimuli can be personalised for the patient.

The overall findings suggest multimodal sensory stimulation can be beneficial for patients, especially those in a minimally conscious state or attempting physical rehabilitation following stroke. Evidence is not strong enough for a recommendation of wide-spread uptake in clinical practice. The research base is limited making it difficult to establish best practice for developing and administering multimodal sensory stimulation. And although negative findings are infrequent in the current literature base, it is unknown if this reflects a publication bias of significant findings. However, from available publications risks appear to be minimal and positive effects common. The evidence base so far suggests future research would be worthwhile.

### Limitations

The small number of studies included in this review makes findings less conclusive. Included studies are generally quite old with only 14 of 38 (36%) occurring in the last decade; more studies took place prior to 2000 (15 of 43). Comparison between studies (including meta-analysis) was made less plausible, and is less reliable, due to the heterogeneity of methods used including senses stimulated, outcome measures used, and dosage (including duration, frequency, and intensity). For example, more than 20 different outcome measures are reported here, from just 25 coma studies. And although it seems the more senses stimulated the better for coma arousal, it is not possible to draw firm conclusions on which sensory modalities are more important; almost all coma studies used auditory, visual, and olfactory as a minimum. Across all studies about 70% of the population are male and for coma studies are aged around 30, for non-coma the average age is much higher at about 53. This homogeneity means results can’t necessarily be applied to populations outside these demographic characteristics. On the other hand, heterogeneity in the state of consciousness between participants at the acute stage, and recovery level at the start and end of the intervention makes comparison and conclusions on when and how long to implement an intervention difficult.

The level of evidence included varies significantly, with the largest number of studies not including a control group, which makes it difficult to compare the reported positive effects to other treatments, or spontaneous recovery. Further, the efficacy of interventions is harder to establish as only 3 identified studies reported effect sizes. Future research should report an effect size.

It is also acknowledged that access to green space for sensory stimulation will not always be practical for many reasons including location of care, stage of recovery etc. Research could explore how to facilitate this access and suggestions include modification of hospital and home internal environments, use of technology such as VR, and increased accessibility to outdoor environments.

### Future Directions

The current paper describes the process for multimodal sensory stimulation as found in academic literature. It is presumed this reflects the practice at the hospitals and rehabilitation units involved. However, it may not fully represent the process in current clinical practice. Further research may shed light on how multimodal sensory stimulation is used in clinical practice, outside of a research design.

Padilla and Domina (2016) conducted a review in 2016 focusing on coma studies and found positive results. The current review finds multimodal sensory stimulation and coma studies in the last five years have not been prevalent; only seven extra papers were found, and most papers since 2016 reported here are no coma studies. Given the promise of multimodal sensory stimulation for increasing arousal during coma further research on this is suggested.

Dosage, including how intense and frequently to administer stimuli, is a priority, especially for patients in a minimally conscious state where evidence for positive changes is consistent but dosage inconsistent. Currently, frequent, small doses of personally relevant stimuli appear to be the most effective approach.

Given the positive findings reported in studies included in this review, it may be worth exploring the use of multimodal sensory stimulation in other injuries and conditions such as PTA, multiple sclerosis, cerebral palsy, or spinal cord injury.

Only one study was identified that explored the effects of injury on behaviours such as agitation, aggression or apathy. This study explored levels of agitation in patients with decreased consciousness after a TBI. Over seven days, an experimental group received auditory and tactile sensory stimulation delivered by a family member, and a control group received routine care. They found no difference between groups in days one to five, but on days six and seven the experimental group experienced significantly lower levels of agitation (Sedghi & Ghaljeh, 2020). Given this positive finding, and the positive effects of multimodal sensory stimulation on these behaviours in other conditions, this would be an interesting and potentially fruitful course of research.

### Conclusion

This review finds studies have been completed in coma and stroke patients. Coma studies measured outcomes in level of consciousness and stroke studies measures motor control and sensorimotor sensations. Multimodal sensory stimulation was adapted so that senses stimulated were appropriate for the outcomes targeted and positive changes are mostly reported. Multimodal sensory stimulation may work better when personalised and made pertinent for the patient; it appears to be a low-risk intervention with positive outcomes.

## Data Availability

No original data collected – material such as data extraction tables available on request.

## Appendix 1. Full Final Search Strategy

The search strategy was developed through a combination of studying past reviews and including other terms of interest. A research librarian assisted in further development of these terms including trialling them for effectiveness.

**Table:**

**Database**	**Limits**	**Search Terms**
Web of Science	Topic, academic	("multi sens*" OR bisens* OR unisens* OR "single sense" OR "sens* stimulation" OR "snoezelen" OR "sens* design*" OR "multi modal sens*" OR "unimodal sens*" OR "bimodal sens*") AND (neurolog* OR "traumatic brain injur*" OR "TBI" OR "acquired brain injur*" OR "ABI" OR "head injur*" OR "brain injur*" OR stroke OR "viral encephal*") AND (relax* OR recove* OR rehabilit* OR intervention OR treatment OR therap*)
CINAHL	Abstract, academic	
ProQuest	Abstract, academic	
PsychInfo	Abstract	
PubMed	All text	

## Appendix 2. Risk of Bias for Included Studies

**Table:**

**First Author (year)**	**Sample Bias**		**Measurement Bias**	**Intervention Bias**		**Overall Direction**
*Coma studies (n* = *28)*						
Abbasi et al. (2009)	(=) Random assignment to treatment or control; No significant difference between control and treatment in level of consciousness (+) Demographics between groups similar, but intervention group significantly more likely to be married (?) Unknown how patients were recruited		(=) Raters were blinded and independent	(=) Nurses were blind to condition (care as usual)		=
Attwell et al. (2019)	(+) Recruitment on a volunteer basis (presumably family/carers)		(=) Raters were blinded and independent; Behaviour grid based on CRS-R	(=) Same tasks indoor as outdoor; Sessions delivered on same day by same therapist; Protocol order randomized (+) Tasks adapted to patients’ abilities		+
Canedo et al. (2002)	(+) Selected case studies		(?) Measurements deemed not useful were discontinued	(+) Co-intervention probable (?) Unknown if same person administered all treatments		+
Deiva et al. (2017)	(=) No significant difference between control and treatment in level of consciousness (?) Demographic differences between the groups unknown		(+) Unknown who collected data – presumably the research team	(=) Unknown if same person administered all treatments – it appears they may have		=
Di Stefano et al. (2012)	(+) Recruitment on a volunteer basis (presumably family/carers)		(=) Same rater each week; Single case ABCBA design (+) Raters were not blinded	(=) Sessions delivered at same times of day (?) Unknown if same person administered all treatments		+
Doman et al. (1993)	(+) Recruitment on a volunteer basis (presumably family/carers) and not random; Control group selected by not wishing to take part in treatment. May have been biased against treatment, been in worst condition, and might not have had same support as treatment group		(?) Unknown who collected data or if they were blinded	(+) Attention bias (?) Unknown if same person administered all treatments		++
Hall et al. (1992)	(+) Recruitment on a volunteer basis (presumably family/carers)		(=) Single case ABAB design (+) Raters were not blinded (therapists were also raters)	(=) Visits of family equal between conditions; Consistency of therapists (+) Probable co-intervention (?) Environmental control not possible		++
Johnson et al. (1993)	(=) Random assignment to treatment or control; Patients similar in age and GCS score at baseline		(=) Some data collection techniques relatively objective (+) Raters were not blinded (therapists were also raters)	(=) Treatment delivered at same time each day (+) Differences in biochemicals and skin conductance within and between groups		+
Kaewsriwong et al. (2015)	(+) Selected case studies – unclear exactly who was excluded; Recruitment on a volunteer basis (family/carers)		(=) Formal training provided to rater’s team for consistency; Same rater each week	(=) Formal training provided to treatment team for consistency (+) Co-intervention not avoided (?) Unknown if treatment took part at same times and days; Unknown if same person administered all treatments		+
Kater (1989)	(=) Demographics between groups not significantly different		(?) Unknown who collected data or if they were blinded	(?) Unknown if treatment took part at same times and days; Unknown if same person administered all treatments		?
Keller et al. (2007)	(+) Recruitment on a volunteer basis (presumably family/carers)		(?) Unknown who collected data or if they were blinded	(+) Co-intervention not avoided; Possible attention bias (?) Unknown if treatment took part at same times and days; Unknown if same person administered all treatments		+
Grüner and Terhaag (2000)	(+) Recruitment on a volunteer basis (presumably by family/carers); No control group or phase (?) Unknown how patients were recruited or selected for the study		(+) One measure introduced after study start (?) Unknown who collected data or if they were blinded	(-) Contamination not avoided (?) Unknown if treatment took part at same times and days; Unknown if same person administered all treatments		+
Megha et al. (2013)	(=) Random assignment to study group; Demographics between groups not significantly different (+) Recruitment on a volunteer basis (family/carers)		(?) Unknown who collected data or if they were blinded	(-) Contamination not avoided (?) Unknown if treatment took part at same times and days		=
Mandeep (2012)	(=) Random assignment to treatment or control (+) Recruitment on a volunteer basis (family/carers)		(?) Unknown who collected data or if they were blinded	(?) Unknown if treatment took part at same times; Unknown if same person administered all treatments		?
Mitchell et al. (1990)	(=) Matched controls; Demographics between groups not significantly different (+) Recruitment on a volunteer basis (family/carers)		(=) Patients families were trained to rate behaviour; inter-rater reliability confirmed (+) Research was not blinded	(?) Unknown if treatment took part at same times and days; Unknown if same person administered all treatments		=
Moattari et al. (2016)	(=) Random assignment to study group; Demographics and level of consciousness between groups not significantly different		(=) Raters were blinded; Ratings took place at same time each day	(=) Treatment delivered at same time each day; it appears treatment was delivered by same family member/nurse each time		=
Noda et al. (2004)	(+) No control group or phase		(?) Unknown who collected data or if they were blinded	(+) Probable attention bias; Probable co-intervention (?) Unknown if treatment took part at same times and days; Unknown if same person administered all treatments		+
Oh and Seo (2003)	(+) Recruitment on a volunteer basis (family/carers)		(=) Single case ABAB design; Rater was blinded to intervention and inter-rater reliability obtained	(+) Probable attention bias; Probable co-intervention (=) Treatment delivered at same time each day in the same order by same researcher		+
Pierce et al. (1990)	(=) No significant differences between control and treatment in demographics (+) Treatment group was on average younger than control		(=) Rater was independent from study and appears to be blinded to intervention (+) Final outcome status not blinded or independent	(=) Treatment delivered at same time each day ( +) Unknown if same person delivered treatment each day within individuals; Co-intervention for treatment group (?) Potential differences in timing and environments between groups		++
Rader et al. (1989)	(?) Unknown how patients were recruited		(?) Unknown who collected data or if they were blinded	(=) Treatment delivered at same time each day (+) Unknown if same person delivered treatment each day; Attention bias; Co-intervention not avoided		++
Sargolzaei et al. (2017)	(=) No significant differences between control and treatment in demographics or level of consciousness		(+) Research was not blinded; Modalities of outcome measure matched the treatment provided (?) Unknown if same person administered all assessments – it appears not	(+) Possible attention bias (?) Unknown if same person administered all treatments – it appears not		+
Talbot and Whitaker, (1994)	(+) Recruitment on a volunteer basis (family/carers) through a relevant association; Families had to agree to be actively present in process		(=) Final evaluation by blinded rater (-) Lowest score for functioning always used	(=) Formal training provided to treatment team for consistency (+) Co-intervention not avoided		+
Urbenjaphol et al. (2009)	(=) Random assignment to treatment or control (+) Recruitment on a volunteer basis (family/carers)		(=) Raters were blinded; Ratings took place at same time each day (?) Unknown if same person administered all assessments	(=) Treatment delivered at same time each day (-) Contamination not avoided (?) Unknown if same person administered all treatments		=
Wijnen et al. (2006)	(+) Recruitment on a volunteer basis (presumably by family/carers); No control group or phase		(=) Rating always took part at same time of day by the same person (+) Research was not blinded	(-) Contamination not avoided (?) Unknown if same person administered all treatments		+
Wilson et al. (1991)	(+) Selected case studies – unclear exactly who was excluded; Recruitment on a volunteer basis (presumably by family/carers)		(+) Unknown who collected data – presumably the research team; Raters unlikely blinded	(-) Contamination not avoided (?) Unknown if treatment took part at same times and days; Unknown if same person administered all treatments		++
Wilson et al. (1993)	(+) Selected case studies – unclear exactly who was excluded; Recruitment on a volunteer basis (presumably by family/carers)		(+) Unknown who collected data – presumably the research team; Raters unlikely blinded	(-) Contamination not avoided (?) Unknown if treatment took part at same times and days; Unknown if same person administered all treatments		++
Wilson et al. (1996)	(+) Selected case studies – unclear exactly who was excluded; Recruitment on a volunteer basis (presumably by family/carers)		(+) Unknown who collected data – presumably the research team; Raters unlikely blinded	(-) Contamination not avoided (?) Unknown if treatment took part at same times and days; Unknown if same person administered all treatments		++
Wood et al. (1993)	(+) No control group or phase (?) Unknown how patients were recruited		(+) Raters were not blinded (?) Baseline measures conducted by different therapists	(-) Contamination not avoided (?) Unknown if treatment took part at same times and days; Unknown if same person administered all treatments		?
*Post-Coma Studies (n* = *15)*						
Gomez et al. (2016)	(=) Demographics between groups not significantly different (+) Recruitment on a volunteer basis		(+) Raters were not blinded	(?) Unknown if same person administered all treatments		=
de Diego et al. (2013)	(=) Random assignment to treatment or control (+) Recruitment on a volunteer basis; Intervention group had less time since lesion occurred		(=) Raters were blinded and independent; Data analyst was blinded and independent	(+) Some therapy for treatment group conducted at home; Attention bias (?) Unknown if treatment/control group actually completed rehabilitation at home		++
Poza et al. (2013)	(=) Demographics between groups not significantly different (+) Recruitment on a volunteer basis		(=) Data collection techniques relatively objective	(=) Some control of contamination stated (?) Unknown if same person administered all treatments; Unknown if treatment took part at same times and days		=
Smania et al. (2003)	(+) Recruitment on a volunteer basis; No control group or phase		(+) Unknown who collected data – presumably the research team	(?) Unknown if same person administered all treatments; Unknown if treatment took part at same times and days		++
Carey et al. (1993)	(+) Selected case studies – unclear exactly who was excluded; Recruitment on a volunteer basis		(+) Unknown who collected data – presumably the research team (?) Outliers removed (unclear which direction they lay)	(?) Unknown if same person administered all treatments		++
Yekutiel and Guttman (1993)	(=) Demographics between groups not significantly different (+) Recruitment on a volunteer basis		(=) It appears the same researcher administered all assessments; Different researcher from treatment delivery rated outcomes (+) Raters were not blinded	(=) The same researcher administered all treatments (?) Unclear if control group were active (potentially creating an attention bias for treatment group), or where they were (potentially creating a site of treatment bias as treatment group were at home)		+
de Jersey et al. (1979)	(+) No control group or phase (?) Patients were not randomly selected		(=) It appears the same researcher administered all assessments (+) Raters were not blinded	(?) Unknown if same person administered all treatments		+
Heine et al. (2017)	(+) Recruitment on a volunteer basis (presumably family/carers); No control group or phase (?) Unknown how patients were recruited		(=) Stimuli order random; Raters were blinded; Inter-rater reliability calculated between raters (high agreement)	(+) Possible co-intervention (?) Unknown if same person administered all treatments		=
Bonan et al. (2016)	(=) Demographics between groups not significantly different (+) Recruitment on a volunteer basis (?) Unknown how patients were selected		(=) Data collection techniques relatively objective (+) Unknown who collected data – presumably the research team	(?) Unknown if same person administered all treatments		=
Byl et al. (2008)	(=) Demographics between groups not significantly different; Some differences between groups at baseline but not statistically significant (+) Recruitment on a volunteer basis by participants who could/would commit to significant involvement and time		(=) Raters were blinded and same rater was used across all studies	(?) Unknown if same person administered all treatments		=
Lynch et al. (2007)	(=) Random assignment to treatment or control (+) Recruitment on a volunteer basis		(=) Raters were blinded; Inter-rater reliability calculated between raters (high agreement) (+) Unclear if same researcher delivered control (relaxation) activities	(=) Both groups received standard physical training (co-intervention)		=
Helliwell (2009)	(+) Recruitment on a volunteer basis; Majority of potential participants excluded due to inadequate communication or cognition resulting in individual case study		(=) Independent rater compared with researcher ratings	(=) Sessions delivered on same day by same therapist (+) Co-intervention		+
Dogru Huzmeli et al. (2017)	(=) Demographics between groups not significantly different (?) Unclear how participants were assigned to control or treatment		(+) Unknown who collected data – presumably the research team	(?) Unclear if treatment took part at same times and days; Unknown if same person administered all treatments		?
Sedghi and Ghaljeh (2020)	(=) Random assignment to treatment or control (+) Volunteered by family members who took part		(+) Unknown who collected data – presumably the research team	(?) Unknown if same person administered all treatments; Unknown if contamination occurred		+
Cheng et al. (2018)	(+) Volunteered by family members who took part		(=) Same rater each week; Raters were blinded and independent; Single case ABAB design	(+) Possible attention bias (-) Contamination not avoided (?) Unknown if same person administered all treatments		=

+ favours treatment group/hypothesis; - favours control group/null hypothesis; = no or negligible effect of bias; ? direction unknown; more than one of + or – signifies a greater effect
